# High Intensity Exercise in Multiple Sclerosis: Effects on Muscle Contractile Characteristics and Exercise Capacity, a Randomised Controlled Trial

**DOI:** 10.1371/journal.pone.0133697

**Published:** 2015-09-29

**Authors:** Inez Wens, Ulrik Dalgas, Frank Vandenabeele, Lotte Grevendonk, Kenneth Verboven, Dominique Hansen, Bert O. Eijnde

**Affiliations:** 1 REVAL Rehabilitation Research Center, BIOMED Biomedical Research Institute, Faculty of Medicine and Life Sciences, Hasselt University, Agoralaan Building A, Diepenbeek, Belgium; 2 Section of Sport Science, Dep. Public Health, Aarhus University, Dalgas Avenue 4, 8000, Aarhus, C, Denmark; Texas A&M University, UNITED STATES

## Abstract

**Introduction:**

Low-to-moderate intensity exercise improves muscle contractile properties and endurance capacity in multiple sclerosis (MS). The impact of high intensity exercise remains unknown.

**Methods:**

Thirty-four MS patients were randomized into a sedentary control group (SED, n = 11) and 2 exercise groups that performed 12 weeks of a high intensity interval (H_IT_R, n = 12) or high intensity continuous cardiovascular training (H_CT_R, n = 11), both in combination with resistance training. M.vastus lateralis fiber cross sectional area (CSA) and proportion, knee-flexor/extensor strength, body composition, maximal endurance capacity and self-reported physical activity levels were assessed before and after 12 weeks.

**Results:**

Compared to SED, 12 weeks of high intensity exercise increased mean fiber CSA (H_IT_R: +21±7%, H_CT_R: +23±5%). Furthermore, fiber type I CSA increased in H_CT_R (+29±6%), whereas type II (+23±7%) and IIa (+23±6%,) CSA increased in H_IT_R. Muscle strength improved in H_IT_R and H_CT_R (between +13±7% and +45±20%) and body fat percentage tended to decrease (H_IT_R: -3.9±2.0% and H_CT_R: -2.5±1.2%). Furthermore, endurance capacity (W_max_ +21±4%, time to exhaustion +24±5%, VO_2max_ +17±5%) and lean tissue mass (+1.4±0.5%) only increased in H_IT_R. Finally self-reported physical activity levels increased 73±19% and 86±27% in H_CT_R and H_IT_R, respectively.

**Conclusion:**

High intensity cardiovascular exercise combined with resistance training was safe, well tolerated and improved muscle contractile characteristics and endurance capacity in MS.

**Trial Registration:**

ClinicalTrials.gov NCT01845896

## Introduction

The heterogeneous symptoms of multiple sclerosis (MS) often lead to a more sedentary lifestyle [1]. This may result in disuse-related loss of exercise capacity and muscle strength, which in turn can affect quality of life [2]. Increasing evidence favors exercise therapy as a method for overall symptom management [3]. Observational [4,5] as well as interventional studies [6–9] have reported improvements in exercise tolerance, muscle strength, functional capacity and health-related quality of life after low-to-moderate intensity cardiovascular or resistance training. Although combined cardiovascular and resistance training could, from a theoretical point of view, positively affect both the cardiovascular system and muscle strength/activation[10], this type of rehabilitation/exercise therapy has not been investigated extensively [11–15].

Several authors already suggested that MS patients could benefit more from higher training intensities [10,16,17], but so far, no studies on combined exercise have evaluated high intensity training in MS. In healthy controls (HC) and in other populations, high intensity exercise and high intensity interval training (H_IT_) have previously been investigated, showing profound improvements in endurance performance and muscle strength [18,19], reduced subcutaneous and abdominal fat [20], improved functional recovery (after stroke) [21] and beneficial effects to the heart [22], emphasising the need to investigate this in MS.

To date the impact of MS on skeletal muscle characteristics, such as muscle fiber cross sectional area (CSA) and proportion remains unclear. Recently, we reported reduced muscle fiber CSA and changed fiber proportions in MS patients, compared to HC [23]. The impact of exercise on muscle contractile properties in MS has only been investigated by Dalgas and co-workers [24]. They reported increased m.vastus lateralis mean fiber CSA combined with improved muscle strength following 12 weeks of progressive resistance training. Despite the importance of understanding the effects of exercise on muscle fiber characteristics to optimize exercise and rehabilitations programs in MS, the impact of other training modalities and intensities on muscle fiber CSA and fiber type proportion in MS, has not been investigated yet.

To determine the effects of high intensity exercise in MS, this study aimed to investigate the impact of high intensity interval or continuous cardiovascular exercise, both in combination with resistance training, on muscle contractile characteristics, in terms of muscle fiber CSA/proportion, muscle strength and muscle mass and on endurance capacity in MS. It was hypothesized that the applied intense programs could improve mean muscle fiber CSA and proportion as well as muscle strength and endurance capacity.

## Methods

### Participants

**Fig 1:**
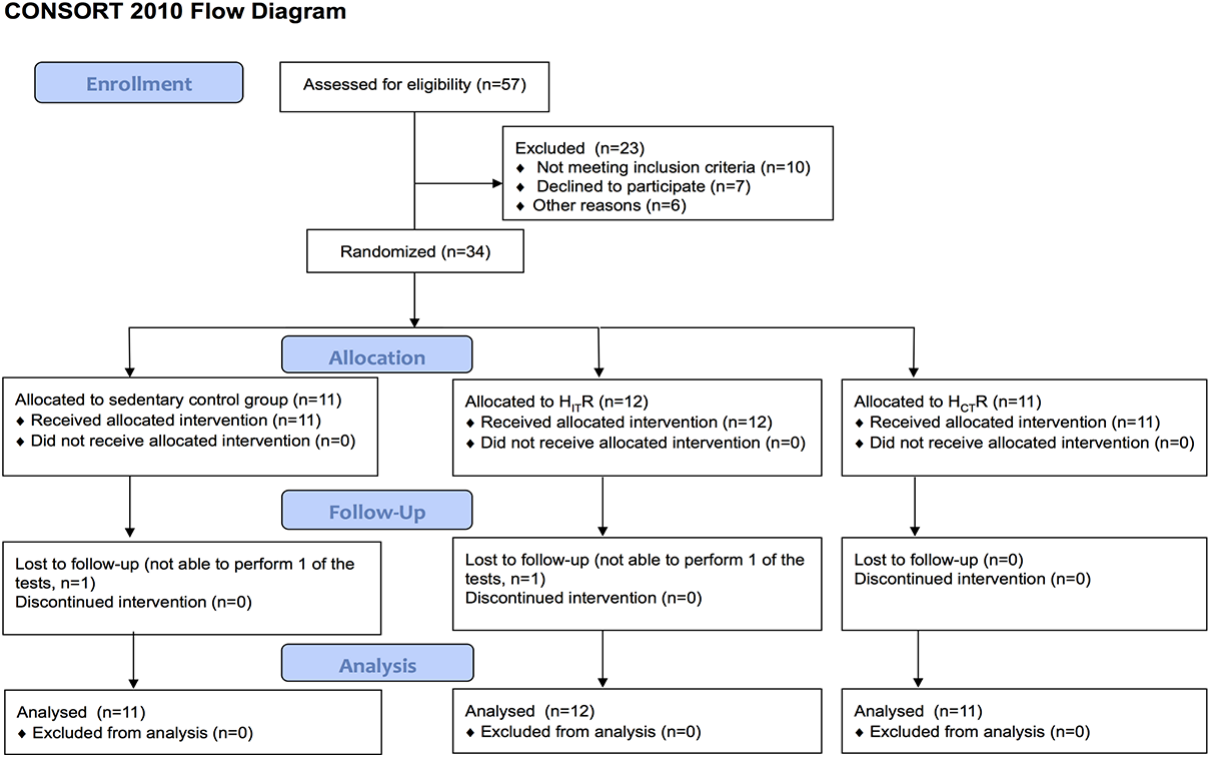
Consort flow diagram for participants’ inclusion.

Thirty-four MS patients diagnosed according to McDonald criteria (EDSS range 1–5), aged >18 years, were included following written informed consent (Fig 1). Subjects were excluded if they had other disorders (cancer, cardiovascular, pulmonary and/or renal), were pregnant, participated in another study, were already physical active, had an acute MS-exacerbation 6 months prior to the start of the study or contra-indications to perform physical exercise.

The study was approved by the ethical committee of Jessa Hospital Hasselt (S1 Protocol) and Hasselt University (12/02/2013), whereupon the preparation of the study started in March 2013 (to order the appropriate equipment, to organise info sessions etc.). Next, this study was registered at ClinicalTrials.gov (NCT01845896, initial release 30/04/2013), at the beginning of patient recruitment (April-June). Furthermore, the authors confirm that all on-going and related trials for this intervention are registered. Finally, all tests were performed in accordance with the Declaration of Helsinki.

### Study design overview

All MS patients were randomized, by means of sealed envelopes, into a sedentary control group (SED, n = 11) and 2 exercise groups that performed 12 weeks of a high intensity interval + resistance training (H_IT_R, n = 12) or high intensity continuous endurance + resistance training (H_CT_R, n = 11). M.vastus lateralis fiber CSA and proportion, knee flexor and extensor strength, body composition, maximal endurance capacity and self-reported physical activity levels were assessed before and after the intervention. Neither the patients nor the researchers involved in the project were blinded to group allocation. SED remained physical inactive during the study course and were instructed to continue their current level of physical activity during the period of the study (S1 CONSORT Checklist).

### Exercise intervention program

After the baseline measurements, the subjects were enrolled in a well-controlled and supervised training program, to increase cardiorespiratory fitness, as well as strength of the major peripheral muscle groups. Subjects participated in 5 sessions per 2 weeks. Training sessions were interspersed by at least one day of rest, to ensure adequate recovery. Each session started with endurance training, followed by resistance training, interspersed by a short resting period.

*H*_*IT*_ *R program*: Each session started with a 5min warm-up on a cycle ergometer. Hereafter, high intensity cycle interval training was performed. During the first 6 weeks exercise duration gradually increased from 5x1min interspersed by 1min rest intervals to 5x2min and 1min rest intervals. Exercise intensity was defined as the heart rate, corresponding to 100% of the maximal workload (which was comparable to approximately 80–90% of the maximal heart rate). During the second 6 weeks, duration remained stable at 5x2min and the heart rate increased to reach a level corresponding to 100–120% of the maximal work load (which was comparable to approximately 90–100% of the initial maximal heart rate). The second part consisted of moderate-to-high intensity resistance training (leg press, leg curl, leg extension, vertical traction, arm curl and chest press, Technogym). In order to exercise at similar relative workload, resistance training of the lower limb was performed unilaterally, due to the frequent bilateral strength differences seen between the legs of MS patients.[25] Training intensity and volume were adjusted from 1x10 repetitions to 2x20 repetitions at maximal attainable load. Maximal attainable load was expressed as the maximal load that the subject was able to manage, under guidance and consequent encouragement. By applying the same standardised encouragements in all groups, subjects were stimulated to perform at their personal maximal ability.

*H*_*CT*_ *R program*: Each session started with a cardiovascular part, consisting of cycling and treadmill walking/running (Technogym). Session duration and exercise intensity increased as the intervention progressed, starting from 1x6min/session to 2x10min/session, at a high workload, corresponding to 80–90% of maximal heart rate and according to individual capabilities. The second part of the training session comprised similar resistance training, as described in the H_IT_R program.

All exercises were performed at a high workload corresponding to 14–16 ratings of perceived exertion on 20-point Borg scale (RPE) and were adjusted to individual disability level. The Borg Rating of Perceiver Exertion Scale measures perceived exertion and is used to document the person’s exertion during a test or to assess the intensity of training and rehabilitation. The scale ranges from 6 to 20, where 6 means “no exertion at all” and 20 means “maximal exertion”. Continuous encouragement by the instructors led to a systematic increase of the training load over the 12-week training period. All sessions were ended by stretching of the extremities, and RPE-level was recorded.

### Primary outcome measure

#### 1. Muscle fiber CSA and proportion

To investigate muscle fiber CSA and proportion, muscle biopsies form the middle part of the m.vastus lateralis (Bergström needle technique) of the weakest leg (see isometric muscle strength measurements) were collected by an experienced medical doctor. The second biopsy, following 12 weeks of exercise or usual care, was taken 2-3cm proximal to the biopsy taken at baseline. Muscle samples were immediately mounted with Tissue-Tek, frozen in isopentane cooled with liquid nitrogen and stored at -80°C, until further analysis. The cross-sections of the biopsies, collected at baseline and after 12 weeks, were processed simultaneously.

Serial transverse sections (9μm) from the obtained muscle samples were cut at -20°C and stained by means of ATPase histochemistry, after preincubation at pH 4.4, 4.6 and 10.3, essentially following the procedure of Brooke and Kaiser [26]. The serial sections were visualized and analyzed using a Leica DM2000 microscope (Leica, Stockholm, Sweden) and a Leica Hi-resolution Color DFC camera (Leica, Stockholm, Sweden) combined with image-analysis software (Leica Qwin ver. 3, Leica, Stockholm, Sweden). A fiber mask of the stained sections was drawn automatically and afterwards this mask was fitted manually to the cell borders of the selected fibers. Only fibers cut perpendicularly to their longitudinal axis were used for the determination of fiber size. On average 170±10 fibers were calculated and included in the CSA and fiber type analyses.

Calculation of the fiber CSA was performed for the major fiber types (I, IIa and IIx) and for the mean fiber CSA, since the number of type IIax and IIc fibers was too small for statistical comparison and CSA calculation.

### Secondary outcome measures

Approximately 1–2 weeks before the muscle biopsy was performed secondary outcome measures were assessed from all subjects.

#### 1. Isometric muscle strength

After 5min of warming-up on a cycle ergometer and following habitation, the maximal voluntary isometric muscle strength of the knee extensors and flexors (45° and 90° knee angle) were measured, as described elsewhere [27], using an isokinetic dynamometer (System 3, Biodex, ENRAF-NONIUS, New York, USA). Two maximal isometric extensions (4s) and flexions (4s), followed by a 30s rest interval, were performed. The highest isometric extension and flexion peak torques (Nm) were selected as the maximal isometric strength. Baseline results were used to classify the legs of each patient as weakest or strongest leg. This subdivision was maintained in further analysis, replacing a conventional left-right classification.

#### 2. Endurance capacity

During the exercise test to volitional fatigue, an electronically braked cycle ergometer (eBike Basic, General Electric GmbH, Bitz, Germany) with pulmonary gas exchange analysis (Jaeger Oxycon, Erich Jaeger GmbH, Germany) was used (cycling frequency: 70 rpm). Jaeger calibration (ambient conditions, volume calibration and O_2_/CO_2_ calibration) was performed at the start of each test day. This test was performed at least 48 hours separated from the isometric muscle strength test to exclude interference of muscle fatigue. Female and male MS patients started at 20W and 30W, respectively, during the first minute. Hereafter, workloads increased, respectively, 10W and 15W per minute. Oxygen uptake (VO_2_), expiratory volume (VE), and respiratory exchange ratio (RER) were collected breath-by-breath and averaged every 10 seconds. Using a 12-lead ECG device, heart rate (HR) was monitored every minute. At the end of the test RER values were evaluated to verify that the test was maximal (RER ≥ 1.15) [28]. In addition, maximal cycling resistance (W_max_), maximal heart rate (HR_max_), test duration and VO_2max_, defined as the corresponding load, heart rate, amount of minutes and oxygen uptake measured at the level of exhaustion, were reported.

#### 3. Body composition

A Dual Energy X-ray Absorptiometry scan (Hologic Series Delphi-A Fan Beam X-ray Bone Densitometer, Vilvoorde, Belgium) was performed pre- en post-intervention. Fat and lean tissue mass were obtained for whole body, legs, trunk, gynoid and android region. Waist-to-hip fat mass ratio (android fat (g)/gynoid fat (g) ratio) and fat mass of the trunk/fat mass of the limbs ratio were calculated.

#### 4. Physical activity level

Before and after the intervention, patients were asked to report their physical activity level by using the Physical Activity Scale for Individuals with Physical Disabilities (PASIPD) [29]. Respondents were asked to report the number of days and average hours in a day spent engaging in 13 activities (including recreational, household, and occupational activities) over the last 7 days. Frequency responses range from 1 (never) to 4 (often), and duration responses range from 1 (less than 1 hour) to 4 (more than 4 hours). Total scores were calculated as the product of the average hours spent in an activity daily and the metabolic equivalents (MET) summed over each item. Scores range from 0 (no activity) to over 100 MET*h/week (very high). At baseline all patients needed to be physical inactive, to be included in the study. Physical inactivity was defined as < 30 MET*h/week.

### Statistical analysis

All data were analyzed using SAS 9.2 software (SAS Institute Inc, Cary, USA). First normality was checked using the Shapiro-Wilk test for all variables. Differences between MS groups (SED, H_CT_R and H_IT_R) were analysed by a one-way ANOVA, whereas within group differences (post minus pre) were analysed with a paired student’s t-test. Relative changes due to the intervention were calculated as the mean of the individual changes and expressed as a percentage. Correlations between changes of the primary and changes of the secondary outcome measures on grouped data from all groups were analysed by means of Pearson’s correlation analysis. Multiple comparison was corrected by means of Bonferroni correction. All data are presented as mean±SE. P<0.01 represents the threshold for statistical significance.

## Results

### Baseline subject characteristics and adherence to the intervention

At baseline, no differences in general subject and disease characteristics (Table 1) as well as outcome measures were found between groups. Approximately 90% of the 30 supervised training sessions were attended in both exercise groups and no severe symptoms exacerbations and/or adverse events were reported. Furthermore, no patient drop out was noted.

**Table 1:** Baseline subject and disease characteristics. Data is presented as mean ± SE. Differences between groups (SED, H_CT_R and H_IT_R) were analysed by a one-way ANOVA. Abbreviations used: MS, multiple sclerosis; SED, sedentary group; H_CT_R, intense continuous endurance + resistance training; H_IT_R, high intensity interval training + resistance training, BMI, body mass index; RR, relapsing remitting; CP, chronic progressive; EDSS, expanded disability status scale; immunomodulatory: interferon β, glatiramer acetate, fingolimod, natalizumab.

	SED (n = 11)	H_CT_R (n = 11)	H_IT_R (n = 12)	p-value
**age (y)**	47±3	47±3	43±3	0.22
**height (m)**	1.67±0.02	1.69±0.02	1.7±0.02	0.32
**weight (kg)**	75.8±3.6	70.2±3.7	75.9±4.1	0.17
**BMI (kg/m^2^)**	27.0±1.4	24.4±1.2	26.1±1.14	0.11
**Lean tissue mass (kg)**	43.2±2.1	45.4±2.6	48.5±3.1	0.11
**Fat percentage (%)**	38.2±2.1	33.6±2.8	36.2±1.9	0.20
**gender (m/f)**	2/9	5/6	5/7	0.12
**type MS (RR/CP)**	8/3	8/3	10/2	0.8
**EDSS**	2.5±0.3	2.7±0.3	2.3±0.3	0.41
**Immunomodulatory MS treatment**	72%	80%	80%	0.23

### Primary outcome measure

#### 1. Muscle fiber CSA and proportion

**Fig 2:**
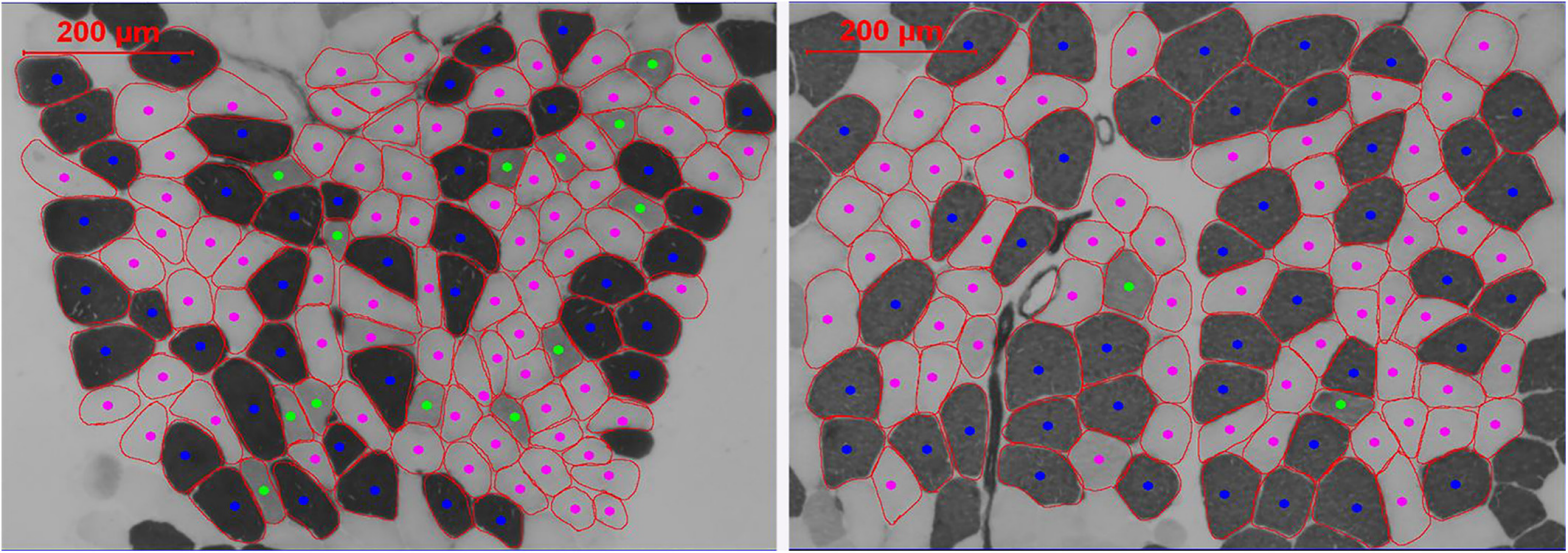
Representative image of fiber type analysis before (left) and after (right) high intensity exercise. Different fiber types are distinguished by color (dark blue: type I, pink: type IIa, green: type IIx, light blue: type IIc). Calculation of the fiber CSA was performed for the major fiber types (I, IIa and IIx) and for the mean fiber CSA, since the number of fibers expressing the minor fiber types (IIax and IIc) was too small for statistical comparison and CSA calculation.

Fig 2 shows a representative image of muscle fiber types before and after high intensity exercise. In SED muscle fiber CSA and proportion did not change (p>0.05). Mean CSA significantly increased in H_IT_R and H_CT_R following 12 weeks of exercise (p = 0.009 and p = 0.002, respectively). Furthermore, muscle fiber type I CSA increased in H_CT_R (p = 0.003), whereas muscle fiber type II and IIa increased in H_IT_R (p = 0.007 and p = 0.002, respectively). Fiber type IIx CSA did not change (p>0.05). In general, no changes in fiber type proportion were observed in any exercise group after 12 weeks of exercise. However, within group effects were observed on type IIx of H_CT_R (p = 0.001), after comparison of the pre- and post-intervention fiber type proportion values (Table 2).

**Table 2:** Muscle fiber type proportion and cross sectional area (CSA) at baseline and after 12 weeks of usual care or high intensity aerobic exercise in combination with resistance training. Data are reported as mean ± SE. Differences between groups (SED, H_CT_R and H_IT_R) were analysed by a one-way ANOVA, whereas within group differences (post minus pre) were analysed with a paired student’s t-test. Relative changes due to the intervention were calculated as the mean of the individual changes and expressed as a percentage. Abbreviations used: SED, sedentary (usual care); H_CT_R, high intensity continuous exercise + resistance training; H_IT_R, high intensity interval training + resistance training.

	SED			H_CT_R			H_IT_R		
	Pre	Post	%	Pre	Post	%	Pre	Post	%
**Fiber type proportion (%)**									
**Type I**	44.2 ± 3.9	47.5 ± 2.9	**7.9 ± 7.5**	40.1 ± 4.7	46.9 ± 4.7^b^	**26.8 ± 11.3**	41.3 ± 3.0	46.3 ± 2.6^b^	**21.7 ± 10.1**
**Type IIa**	34.2 ± 3.9	34.2 ± 2.3	**5.1 ± 13.1**	34.1 ± 2.9	38.9 ± 4.6	**6.6 ± 7.5**	40.9 ± 3.8	44.5 ± 2.4	**6.9 ± 8.1**
**Type IIx**	21.2 ± 4.5	17.7 ± 2.0	**19.2 ± 12.6**	24.3 ± 2.7	13.5 ± 2.6^a^	**-46.0 ± 7.6** ^c^	18.5 ± 2.8	10.1 ± 2.8	**-20.1 ± 25.4**
**Fiber CSA (μm^2^)**									
**Mean**	3738 ± 267	3740 ± 431	**3.5 ± 4.3**	3551 ± 351	3905 ± 408^a^	**23.3 ± 4.9** ^c^	4038 ± 321	4892 ±379^a^	**21.1 ± 7.3** ^d^
**Type I**	4078 ± 384	4050 ± 531	**4.0 ± 5.5**	3630 ± 443	4071 ± 470^a^	**29.8 ± 5.5** ^c^	4410 ± 188	4916 ± 399	**12.1 ± 8.7**
**Type II**	3487 ± 265	3478 ± 334	**6.9 ± 5.8**	3285 ± 321	3622 ± 398^b^	**20.8 ± 7.9**	3612 ± 429	4551 ± 462^a^	**22.7 ± 6.8**
**Type IIa**	3703 ± 306	3729 ± 402	**3.6 ± 3.1**	3719 ± 366	4014 ± 522^b^	**15.1 ± 5.3**	4037 ± 444	5034 ± 447^a^	**22.8 ± 6.2** ^d^
**Type IIx**	3446 ± 305	3191 ± 318	**5.4 ± 8.2**	2771 ± 277	2955 ± 258	**14.5 ± 8.9**	3187 ± 438	3920 ± 519^b^	**23.6 ± 8.8**

^a^ p<0.01

^b^ p ≤ 0.05, compared with pre-intervention value, within group.

^c^ p<0.01

^d^ p ≤ 0.05, pre to post change compared with change from pre to post in SED.

### Secondary outcome measures

#### 1. Isometric muscle strength

**Fig 3:**
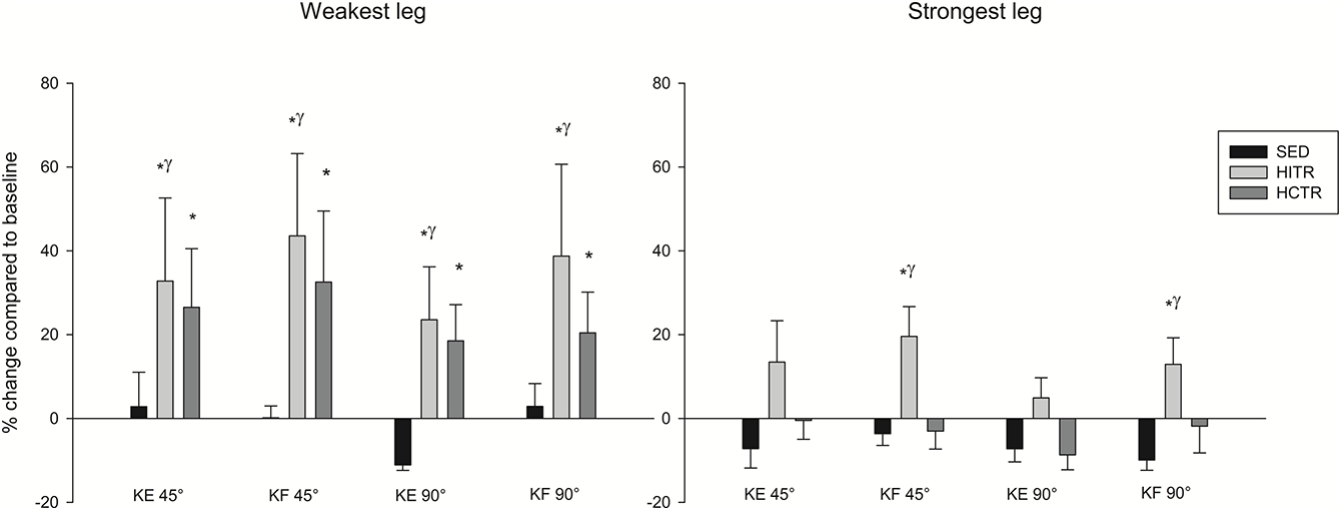
Percentage change of knee extension and flexion after 12 weeks of physical inactive living (usual care, SED), high intensity continuous training + resistance training (H_CT_R) and high intensity interval training + resistance training (H_IT_R). Data are reported as mean ± SE. * p<0.05, compared with pre-intervention value, within group. ˠ p<0.05, pre to post change compared with change from pre to post in SED. Abbreviations used: KF, knee flexion; KE, knee extension.

Muscle strength of SED remained stable during 12 weeks of usual care (p>0.05, Fig 3). Compared to SED, knee flexion and knee extension strength of the weakest leg of H_IT_R improved by 24±13 to 44±20% (p values between 0.01 and 0.006), whereas only hamstring strength of the strongest leg of H_IT_R improved by 13±7 to 20±7% (p = 0.006). Furthermore, H_CT_R flexion and extension strength improved, from pre- to post trial, in the weakest leg by 19±9 to 33±17% (p values between 0.01 and 0.006), whereas muscle strength of the strongest leg remained stable (p>0.05).

#### 2. Endurance capacity

After 12 weeks, endurance capacity variables remained stable in SED and H_CT_R. Compared to SED and H_CT_R, W_max_ (+21±4%, p = 0.0001), test duration (+24±5%, p = 0.00008) and VO_2max_ (+17±5%, p = 0.001) significantly improved in H_IT_R (Table 3).

**Table 3:** Exercise capacity, body composition and physical activity level after 12 weeks of usual care or high intensity aerobic exercise in combination with resistance training. Data are reported as mean ± SE. Differences between groups (SED, H_CT_R and H_IT_R) were analysed by a one-way ANOVA, whereas within group differences (post minus pre) were analysed with a paired student’s t-test. Relative changes due to the intervention were calculated as the mean of the individual changes and expressed as a percentage. Abbreviations used: SED, sedentary (usual care); H_CT_R, high intensity continuous exercise + resistance training; H_IT_R, high intensity interval training + resistance training; MET, metabolic equivalent.

	SED			H_CT_R			H_IT_R		
	Pre	Post	%	Pre	Post	%	Pre	Post	%
**Exercise capacity:**									
**Maximal cycling resistance (watt)**	121±8	115±11	**-4.6±2.7**	131±18	133±18	**3.6±2.8**	158±15	188±15^a^	**21.2±3.9** ^c^
**Maximal cycling resistance (watt/kg)**	1.6±0.12	1.6±0.15	**-4.6±2.7**	1.85±0.24	1.9±0.23	**3.6±2.8**	2.0±0.17	2.4±0.16^a^	**21.2±3.9** ^c^
**Test duration (min)**	10.4±0.8	9.9±0.8	**-3.1±2.9**	9.5±1.0	9.8±0.9	**5.2±3.1**	12.1±0.9	14.5±0.9^a^	**24.7±4.6** ^c^
**VO**_**2**_ **max (ml/min)**	1647±133	1645±160	**2.5±4.1**	1870±238	1969±230	**7.5±5.8**	2031±186	2379±197^a^	**17.8±4.6** ^c^
**VO**_**2**_ **max (ml/min/kg)**	21.9±1.8	23.6±2.1	**2.5±4.1**	26.3±3.1	28.2±3.0	**7.5±5.8**	26.6±2.2	30.7±2.1^a^	**17.8±4.6** ^c^
**Minute Ventilation (l/min)**	57±4	62±7	**9.9±6.5**	70±11	76±11^b^	**13.3±7.7**	76±7	96±6^a^	**32.7±8.7**
**Breathing frequency**	32±2	39±3^a^	**25.7±5.5**	32±2	37±2^a^	**14.3±4.6**	32±2	41±3^a^	**39.6±16.8**
**Tidal Volume (ml)**	1789±138	1617±154	**-11.2±6.2**	2155±241	2086±287	**-1.2±4.6**	2394±190	2425±189	**-0.5±5.2**
**RER max**	1.18±0.04	1.17±0.03	**-3.2±2.8**	1.3±0.03	1.2±0.02	**-2.2±2.9**	1.2±0.03	1.2±0.02	**1.3±2.5**
**HR rest (beats/min)**	75±4	87±4^a^	**14.3±3.8**	76±3	80±4	**7.0±5.8**	75±3	84±3	**12.5±4.6**
**HR max (beats/min)**	142±7	153±5	**6.5±2.3**	154±6	162±6^b^	**3.7±1.5**	160±6	168±5^a^	**6.2±2.2**
**Body composition:**									
**Lean tissue mass (kg)**	43.2±2.1	43.5±2.1	**0.6±0.6**	45.4±2.6	46.2±2.5	**0.9±0.9**	48.5±3.1	49.9±3.1^a^	**1.4±0.5**
**Fat percentage (%)**	38.2±2.1	37.3±2.2	**-2.8±1.6**	33.6±2.8	32.6±2.8^b^	**-2.5±1.2**	36.2±1.9	34.3±2.0^b^	**-3.9±2.0**
**Physical activity level:** (MET*h/week)	16±2.6	15.8±3.7	**2.9±13**	14.7±2.7	23.9±4.4^a^	**73±19** ^c^	25.8±6.6	37.6±7.2^a^	**86±27** ^c^

a p<0.01,

b p<0.05, compared with pre-intervention value, within group.

c p<0.01, pre to post change compared with change from pre to post in SED.

#### 3. Body composition

Following 12 weeks of exercise, body weight remained stable in all groups (p>0.05). Within H_IT_R and H_CT_R, body fat percentage tended to decrease by 3.9±2.0% (p = 0.04) and 2.5±1.2% (p = 0.02), respectively. Furthermore, lean tissue mass significantly increased 1.4±0.5% within H_IT_R (p = 0.01), whereas it remained stable in H_CT_R and SED (p>0.05, Table 3). Finally, other adipose and lean tissue mass indices remained stable in all groups (p>0.05).

#### 4. Physical activity level

Compared to SED, the physical activity level of H_IT_R and H_CT_R significantly increased by 86±27% (p = 0.004) and 73±19% (p = 0.003), respectively, following 12 weeks of exercise. In SED the physical activity level remained stable (Table 3).

### Correlations

Overall, no significant correlations were found between the change of the primary and secondary outcome measures on pooled data.

## Discussion

This study is the first to investigate the impact of high intensity cardiovascular exercise combined with resistance training on muscle contractile characteristics and endurance capacity in MS. Moreover, 12 weeks of the applied high intensity programs were safe, well tolerated and induced beneficial adaptations in MS patients. In particular, muscle fiber CSA, muscle strength of the weaker legs and self-reported physical activity levels improved following both H_IT_R and H_CT_R. In addition, further improvements of the endurance capacity, muscle flexion strength of the stronger legs and lean tissue mass were only seen in H_IT_R. These results are clinically relevant, due to the need for exercise programs that are able to counteract reduced endurance capacity, muscle strength and muscle mass of particularly the lower limbs, enhancing physical function in MS patients.

### Safety and tolerability

Several studies have already demonstrated the benefits of resistance training [6] or endurance training [7–9] in MS. The effect of combined training has only been sparsely explored [11–14] and the impact of high intensity combined exercise has never been investigated before. The latter could be explained by safety concerns regarding the symptom instability of MS patients often seen during/after high intensity exercise, which is frequently caused by the exercise-induced increase in body temperature [30]. Interestingly, no dropout or adverse events were reported during and after 12 weeks of H_IT_R and H_CT_R, demonstrating that mild-to-moderately impaired MS patients tolerate intense exercise programs.

### Continuous vs. interval training

The present study showed an improvement of the endurance capacity, muscle flexion strength of the stronger legs and lean tissue mass in H_IT_R, and improved muscle strength of the weaker leg and self-reported physical activity levels in H_IT_R and H_CT_R, suggesting that exercise efficiency is even higher in H_IT_R. This is in line with literature in other patient populations, investigating the difference between continue and interval training, stating that exercise intensity is an important factor to improve, amongst others, cardiorespiratory fitness [31–33], but also arterial stiffness [34] and hypertension [35]. In general, the magnitude of improvements was greater after high intensity interval training. Importantly, and as already suggested by others [10], the observed training improvements in the present study were often larger compared to those reported after mild-to-moderate combined exercise programs in MS patients [11–15]. This suggests that higher training intensities are more effective and that training adaptations are intensity related in MS.

Interestingly, the maximal heart rate changed from baseline to post training in H_IT_R. This can possibly be explained due to the fact that these patients might have impaired chronotropic regulation at baseline, which can broadly be defined as the inability of the heart to increase its rate commensurate with increased activity or demand, which might be induced by cardiac autonomic dysfunction, as already reported by our research group [36,37]. In other populations, exercise is able to increase peak heart rate and to reverse, at least partially, impaired chronotropic regulation [38–42], which contribute to the exercise-induced increase in exercise capacity and other outcome measures. Since this was only seen in H_IT_R and not in H_CT_R, it suggests again that higher training intensities might be more effective in MS. Nevertheless, impaired chronotropic regulation was never investigated into depth in MS patients and warrants further research in the future.

### Muscular effects

Recently, we reported that MS affects muscle fiber CSA and proportion [23]. To our knowledge, only Dalgas et al. investigated the effects of exercise (progressive resistance training) on muscle fiber CSA in MS [24], reporting increased mean muscle fiber CSA (8±15%), predominantly in type II muscle fiber CSA (14±19%) and a tendency towards increased type I CSA [24]. In the present study, mean muscle fiber CSA (H_IT_R: 21±7%, H_CT_R: 23±5%) and lean muscle mass further increased, suggesting an additional value of the high intensity aerobic exercise. This is, partly, in accordance with results reported in sedentary HC, demonstrating a significant increase of the area of type I and IIx fibers after high intensity interval training [43]. In addition, high intensity aerobic exercise induced an increased CSA of both type IIa and IIx fibers and no changes in type I fiber size in elite ice hockey players [44].

Based on an often more inactive lifestyle of MS patients, Dalgas et al. expected an inactivity-related higher proportion of type IIx fibers and a possibility to transform type IIx to IIa fibers after progressive resistance training [45,46]. However, they were not able to report any changes in the proportion of fiber types. In the present study, type IIx proportions decreased after 12 weeks of H_CT_R, whereas the type I proportion tended to increase in H_CT_R and H_IT_R. These results are comparable with data reported in healthy elderly populations, reporting a reduction of the type IIx proportion and an increase of the proportion of the type IIa fibers [47,48]. Interestingly, these studies used higher training frequencies [47] or longer training periods [48], compared to the work of Dalgas et al. [24], suggesting that a higher training volume and intensity is required to induce fiber type changes than to induce changes in fiber type CSA.

### Limitations

Since this is the first study that investigated the effects of high intensity exercise on muscle fiber CSA and proportion in MS, we were not able to perform a pre-trial power analysis, due to the absence of a defined effect size. Nevertheless, a post-hoc power analysis (R 2.15.2 software) on mean muscle fiber CSA and based on the present results, demonstrated that 5 persons in each group would be sufficient to provide a >80% power to detect a 20% increase of mean muscle fiber CSA after 12 weeks of high intensity exercise (p = 0.05, σ = 7%), demonstrating a suitable sample size in the present study. Secondly, given the ethical concerns we collected only one biopsy per test, despite the recommendation of Lexell et al. [49] to optimally collect three biopsies from different depths of the muscle and to analyse >150 fibers from each sample to reduce sampling error. Furthermore, since self-reported physical activity measures are not perfect measures, we propose the use of accelerometers in future studies. Also the inclusion of a follow up examination, to determine whether the improvements are long lasting, could be recommended in future studies. Finally, given the nature of the design, social interactions between MS patients could possibly influence intervention outcomes.

## Conclusion

The present study showed that 12 weeks of high intensity cardiovascular exercise in combination with resistance training was safe, well tolerated and improved muscle contractile characteristics and endurance capacity, with interval training seemingly superior to continuous training.

## Supporting Information

S1 CONSORT Checklist CONSORT Checklist. (DOC)

S1 Protocol Trial Protocol. (DOCX)
