# Supplementary material for: High Intensity Exercise in Multiple Sclerosis: Effects on Muscle Contractile Characteristics and Exercise Capacity, a Randomised Controlled Trial
Source: PLoS One. 2015 Sep 29;10(9):e0133697. doi: 10.1371/journal.pone.0133697 (PMC4587912; doi:10.1371/journal.pone.0133697)
Supplement: S1 Protocol — (DOCX) [file pone.0133697.s002.docx]

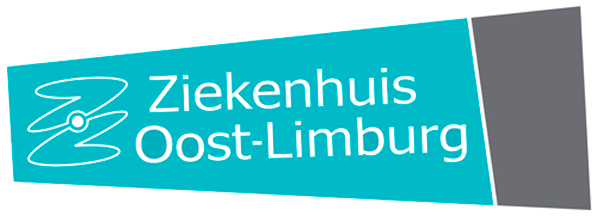

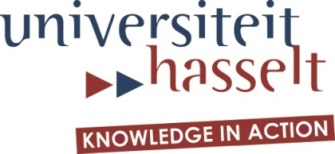

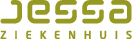


+32 89 32 13 25

[ec.submission@zol.be](mailto:ec.submission@zol.be)

+32 11 26 85 02

[CME @uhasselt.be](mailto:marleen.missotten@uhasselt.be)

+32 11 30 90 06

[ethische.toetsingscommissie@jessazh.be](mailto:ethische.toetsingscommissie@jessazh.be)


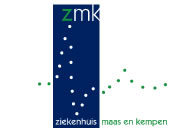


+32 89 50 99 52 [c.goossens@zmk.be](mailto:c.goossens@zmk.be)


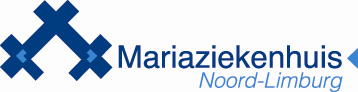


+32 11 82 64 48

[mtheuws@mznl.be](mailto:mtheuws@mznl.be)


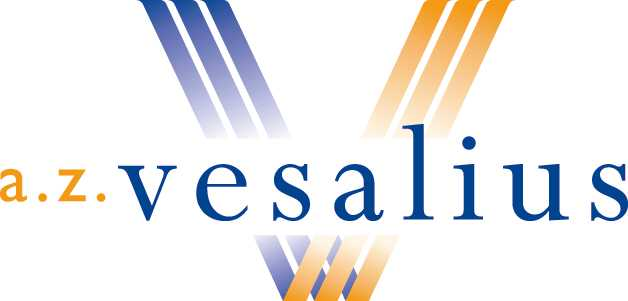


+32 12 39 61 11

[CME@azvesalius.be](mailto:CME@azvesalius.be)


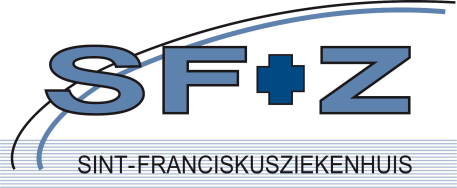

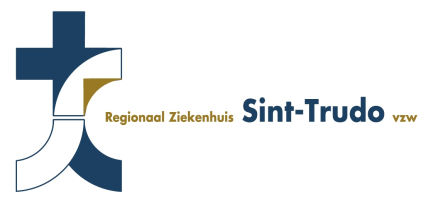


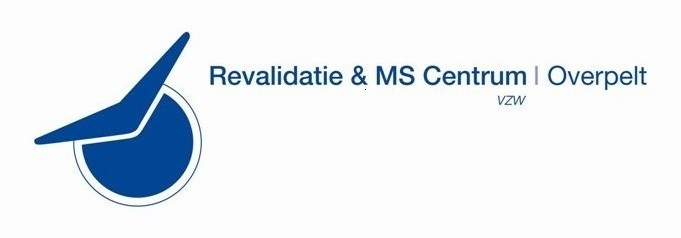


+32 11 82 64 48

[mtheuws@mznl.be](mailto:mtheuws@mznl.be)

+32 475 34 28 17

[ethische.commissie@sfz.be](mailto:ethische.commissie@sfz.be)

+32 11 69 96 00

[Jolanda.Verheezen@rzst.be](mailto:Jolanda.Verheezen@rzst.be)

# Application form ethical committee

# for experiments on humans

***1. Summary***

Multiple sclerosis (MS) is the most frequently common neurological disease in western adults. This auto-immune disease is characterized by a variation of symptoms (tremor, spasticity, partial paralysis) caused by demyelination and axonal atrophy in the central nervous system (CNS)^1^. Consequently, this leads to a more sedentary lifestyle in MS patients. Furthermore, this results in loss of functional strength and exercise capacity^2^ and an elevated risk to develop secondary health complications^3, 4^.

Several studies in MS reported positive effects of physical activity on the course of this disease. Specifically, the effect of physical activity in this population is multilayered. In recent years, several studies have demonstrated the effectiveness of physical exercise programs to influence muscle strength and endurance in MS patients^5, 6^ positively without worsening of MS related symptoms as fatigue^7^. Currently, it is relatively unclear which exercise modalities should be considered as optimal (endurance and/or strength training) to acquire functional benefits in both strength and endurance capacity in MS patients, and is mild to moderate physical activity being advised for this population^2^.

Interestingly, recent research in EAE rats, the animal model for MS, has demonstrated that intense physical activity, compared to mild and moderate intensity, would be the most optimal exercise intensity. Intense physical activity causes retardation of the disease process, though the further course of this disease would not be changed by it^8-12^. Furthermore, it turns out that body weight of the intense exercise group was higher compared to other groups, which potentially could point out to better general health conditions (unpublished data). Therefore, the influence of more intense physical activity in persons with MS should be investigated.

The loss of functional strength and appearance of general muscle weakness in MS, is probably caused by changes in contractile characteristics, such as muscle fiber morphology and composition. Due to the limited amount of studies regarding this domain, it is however yet unclear which alterations occur in muscle fiber composition following MS^13, 14^. Research concerning the influence of physical activity on muscle characteristics in MS patients is, as indicated previously, scarcely available. Recent investigation though demonstrated significant improvements in muscle fiber cross-sectional area and a significant increase in muscle strength subsequent to resistance training^15, 16^. The muscle fiber proportion on the other hand did not change^15^. The influence of a combined (endurance and resistance) exercise program, nor the effect of high intensity interval training (HIIT) on muscle characteristics in persons with MS was yet investigated.

Finally, several studies displayed that physical activity could prevent impaired glucose tolerance (IGT), and the accompanying type 2 diabetes in other populations. Moreover, physical therapy was often used as the primary treatment against the development of IGT in patients with metabolic syndrome or other diseases^17, 18^. The influence of a combined (endurance and resistance) exercise program on glucose tolerance in MS patients, was recently investigated for the first time, but displayed no alterations in this study^19^. Although the positive effect of more intense physical activity and HIIT training was already reported on glucose tolerance in other populations, this was never investigated in persons with MS.

The purpose of this research is plural:

1. We want to examine the effect of a 12-week continuous aerobic exercise or HIIT exercise, both in combination with resistance training, on muscle fiber characteristics and metabolism and explore the extent to which high intensity exercise influences exercise capacity, muscle strength and body composition in persons with MS.
2. Moreover, based on the findings that a combined exercise program does not affect the occurrence of glucose tolerance in persons with MS (previous research^19^), we want to examine whether a HIIT training program could display another influence on the development of glucose intolerance in persons with MS.

***2. Study design***

*Subjects:*

Thirty-six MS patients and 18 healthy controls (HC) will participate in this study, obtaining a 2:1 ratio. Inclusion criteria on admission will be: male or female, older than 18 years and, in case of the MS patients, physician-diagnosed MS (EDSS score between 0,5-6). Subjects will be excluded if they have physician-diagnosed diabetes mellitus type II, have other disorders, are pregnant, participate in another study, have, in case of the MS patients, contra-indications to perform physical exercise or had an acute MS exacerbation 6 months prior to the start of the study. The study is submitted to be approved by the ethical committee, will be performed in accordance with the Declaration of Helsinki. Written informed consent to participate in the study will be obtained from all participants.

*Study design:*

At baseline, all MS patients and HC will undergo a muscle biopsy. Next all MS patients will perform an oral glucose tolerance test (OGTT) to determine glucose tolerance. Muscle strength and endurance capacity of the MS patients will be measured as secondary outcome measures, using a muscle performance protocol on the Biodex and a maximal endurance protocol on an ergometer, respectively. Hereafter, MS patients will be divided into a sedentary control group (SED) and 2 exercise groups, a high intensity interval + strength training group (HIIT) and an intense combined (continuous endurance + strength) exercise group (COMB). Patients of the exercise groups will be enrolled in an exercise program during 12 consecutive weeks. Here subjects perform five training sessions per two weeks with at least 48 hours of rest between training sessions. The SED group will not participate in any training program. They will be asked to continue their current level of physical activity during the period of the study and not to engage in any form of exercise. To evaluate the impact of the intervention program test will be repeated after 12 weeks.

*Exercise program:*

**Combined program:** Each session will start with a cardiovascular part, consisting of cycling and treadmill walking or running (Technogym®). Session duration and exercise intensity increases as the intervention progresses, starting from 1x6 min/session to 2x10 min/session, at a high workload, corresponding to 80-90% of maximal heart rate and according to individual capabilities. The second part consists of resistance training (leg press, leg curl, leg extension, vertical traction, arm curl and chest press, Technogym®). Resistance training of the lower limb will be performed unilaterally, due to bilateral strength differences between the legs of MS patients. To improve maximal power as well as to reduce muscle work fatigue, exercise workloads and sets of repetitions gradually increases during intervention, from 1x10 repetitions to 2x20 repetitions, at a moderate to high intensity workload corresponding to 12-14 ratings of perceived exertion on 20-point Borg scale and according to individual capabilities.

**HIIT program:** Each session starts with a 5min warming up (cycling). During the first 6 weeks exercise duration gradually increases from 5x1min (1min rest intervals) to 5x2min (1min rest intervals) at a high intense workload, corresponding to 100% of the maximal power (watt) and 100% of the maximal heart rate, where after, in the second 6 weeks of the program, duration remains stable and workload increases to reach 100-120% of the maximal workload. The second part of the training session comprises resistance training, as described in the combined program.

*Oral glucose tolerance test (OGTT):*

The glucose profile of MS patients will be investigated using an OGTT, including determination of blood glucose and serum insulin levels throughout the OGTT. At 8 a.m., after a 10h overnight fasting period, all participants will receive a 1g glucose/kg bodyweight glucose bolus. Capillary blood samples, to immediately measure whole blood glucose concentrations (Analox GM7 Micro-stat, Analox instruments Ltd, London, UK), will be collected from a hyperaemic earlobe, before and after glucose administration, at 20min intervals during a 2h period. To determine serum insulin levels, 4 cc of venous blood will be collected in serum separation tubes (SST, BD Vacutainer®, Becton-Dickinson, Erembodegem, Belgium) at 1h intervals. After 30min, allowing blood clotting, samples will be centrifuged during 10min on 3500rpm. Thereafter, the obtained serum will be frozen and stored at -80°C until batch analysis of serum insulin levels, according to manufacturer’s instructions, will be performed (Mercodia Insulin ELISA, Uppsala, Sweden).

*Isometric muscle strength:*

The maximal voluntary isometric and dynamic muscle strength of the knee extensor and flexor will be measured by means of an isokinetic dynamometer (System 3, Biodex, ENRAF-NONIUS, New York). After 5 minutes of warming up and habitation, the maximal isometric muscle strength of the knee extensor and knee flexor will be measured (knee at 45° and 90°), by performing 2 maximal isometric extensions (4 sec) and flexions (4 sec), followed by a 30 seconds rest interval. The highest isometric extension and flexion (Nm) will be selected as the maximal isometric strength. At baseline, the legs of each patient will be subdivided in a “weak” and a “strong” leg. This classification will be used for further analysis, instead of a “left-right” analysis.

*Maximal test on a cycle ergometer:*

During the exercise test to volitional fatigue an electronically braked cycle ergometer (eBike Basic, General Electric GmbH, Bitz, Germany) with pulmonary gas exchange analysis (Jaeger Oxycon, Erich Jaeger GmbH, Germany) will be used (cycling frequency: 70 rpm). Male MS patients will start at 30W the first minute, where after the load increases 15W per minute. Female MS patients will start at 20W and increases 10W per minute. Oxygen uptake (VO2), expiratory volume (VE), and respiratory exchange ratio (RER) will be collected breath-by-breath and averaged every 10 seconds. Using a 12-lead ECG device, heart rate (HR) will be monitored and lactate concentrations will be measured every minute. In addition, maximal cycling resistance (Wmax) will be reported.

*Body composition:*

A Dual Energy X-ray Absorptiometry scan (GE Hologic Series Delphi-A, Vilvoorde, Belgium) will be performed. Fat and lean tissue mass will be obtained for the whole body as well as for different regions covering the legs, the trunk, the gynoid and the android region. Waist-to-hip fat mass ratio (android fat (g)/gynoid fat (g) ratio) and fat mass of the trunk/fat mass of the limbs ratio will be calculated.

*Muscle biopsy:*

Muscle biopsies will be obtained, by an experienced blinded medical doctor, from the middle part of the m.vastus lateralis by means of a Bergström needle ([Dietrichson, Coakley et al. 1987](#_ENREF_10)). The biopsy at the end of the study will be taken 2-3cm proximal to the biopsy taken at baseline. The collected tissue will be immediately frozen and stored at -80°C, until further analysis will be performed.

*Muscle fiber analysis:*

Serial transverse sections (9µm) from the obtained muscle samples will be cut at -20°C and stained by means of ATPase histochemistry, after preincubation at pH 4.4, 4.6 and 10.3, essentially following the procedure of Brooke and Kaiser^20^. The serial sections will be visualized and analyzed using a Leica DM2000 microscope (Leica, Stockholm, Sweden) and a Leica Hi-resolution Color DFC camera (Leica, Stockholm, Sweden) combined with image-analysis software (Leica Qwin ver. 3, Leica, Stockholm, Sweden). This software is able to automatically draw a fiber mask at the stained sections. Afterwards, this mask is fitted manually to the cell borders of the selected fibers. Only fibers cut perpendicularly to their longitudinal axis will be used for the determination of fiber size.

*GLUT4 and glycogen analysis:*

Glycogen content will be determined by a standard enzymatic fluorometric assay^21^. GLUT4 measurements will be performed with Western blot analysis. In brief, 20–30 mg of wet muscle will be homogenized (Polytron) on ice in a buffer (pH 7.4) with the following composition: 250 mmol/l sucrose, 20 mmol/l NaHCO3, 1 mmol/l phenylmethylsulfonyl fluoride, 0.5% nonionic surfactant (Triton X-100), and a protease inhibitor cocktail tablet (Complete, Roche). The homogenate will then be incubated under gentle agitation at 4°C for 30 min and spin for 15 min at 13,000 g (4°C). The protein concentration of the supernatant will be determined by using a BCA protein assay kit (Pierce). Then, 45g of supernatant protein will be separated by SDS-PAGE with 10% Tris-glycine polyacrylamide gels. After the proteins will be transferred to a polyvinylidene fluoride membrane, the membrane will be blocked at room temperature for 1 h in TBS-T (Tris-buffered saline containing 0.05% Tween 20, pH 7.4) comprising 5% skimmed milk powder. Blocked membranes will be incubated overnight at 4°C in TBS-T comprising 1% skimmed milk powder and a specific rabbit polyclonal antibody against 13 COOH-terminal amino acids of GLUT4 (AB1346, Chemicon International, Temecula, CA). Finally, GLUT4 will be visualized with an alkaline phosphatase-conjugated goat anti-rabbit secondary antibody (DAKO, Copenhagen, Denmark) by fluorescence imaging and band density will be calculated by using image analysis software. To correct for intergel differences, band intensity will be expressed relative to a standard that will run together with the samples.

***3. Expected benefits for the participant and/or science***

Participants in the intervention group of this study receive the possibility to improve their general health and physical fitness, accompanied by a team of professionals. Moreover, glucose intolerace or diabetes could be detected in an early phase of the disease, by means of an oral glucose tolerance test, which could benefit remediation of this disease. Furthermore, all participants provide a selfless contribution to science and the search for new/improved rehabilitation techniques and training programs for persons with MS. Finally, the donation of muscle tissue provides the possibility to further investigate the muscle characteristics in MS. Since comparable research is limited, this study will be relevant for science in general, and more specifically for the research in MS.

***4. Evaluation of the predictable treatment risks and/or procedures (pain, discomfort, invasive treatment, measures to diminish these risks and undesirable effects of the procedures, possible contact with the researcher,…)***

This investigation and measurements will be executed in such a way, it will not entail important risks. Potential discomforts for the patient could be:

- Dizziness, faint, hypotension because of the fasting period prior to the oral glucose tolerance test.
- The collection of blood samples does not contain important risks. The patient could though experience slight pain or discomfort by the needle, which could result into light nausea or a small hematoma.
- Certain participants could experience nausea after drinking the glucose mixture prior to the glucose tolerance test. This should normally disappear after 2 hours.
- Measuring height and weight (to determine BMI) does not contain risks or discomforts.
- DXA-scans operate with only small quantities of radiation. No special measures are considered.
- Collections of muscle biopsies by a medical doctor can be slightly painful, though harmless. No permanent damage is applied to the muscle.
- All participants will receive an individual based training program and are accompanied by experienced physiotherapists. Slight stiffness of the trained muscle groups could occur, which will disappear after habituation to the more intense training sessions.

Should certain complications occur during the study, participants can make use of an insurance which is arranged by the research team. If problems do arise, the participants are requested to contact a member of the research team (Dr. Inez Wens: 0032 (0) 11/26.93.70 or Prof. Dr. Bert Op’t Eijnde: [bert.opteijnde@uhasselt.be](mailto:bert.opteijnde@uhasselt.be)).

Reference List

(1) Noseworthy JH, Lucchinetti C, Rodriguez M, Weinshenker BG. Multiple sclerosis. *N Engl J Med* 2000;343(13):938-952.

(2) Dalgas U, Stenager E, Ingemann-Hansen T. Multiple sclerosis and physical exercise: recommendations for the application of resistance-, endurance- and combined training. *Mult Scler* 2008;14(1):35-53.

(3) Wens I., Dalgas U., Deckx N., Cools N., Eijnde BO. Does Multiple sclerosis affect glucose tolerance? *Mult Scler* 2013;20(9):1273-1276.

(4) Wens I., Dalgas U., Stenager E., Eijnde B.O. Risk factors related to cardiovascular diseases and the metabolic syndrome in multiple sclerosis - a systematic review. *Mult Scler* 2013;19(12):1556-1564.

(5) Motl RW, McAuley E, Snook EM. Physical activity and multiple sclerosis: a meta-analysis. *Mult Scler* 2005;11(4):459-463.

(6) Motl RW, Gosney JL. Effect of exercise training on quality of life in multiple sclerosis: a meta-analysis. *Mult Scler* 2008;14(1):129-135.

(7) Dalgas U, Stenager E. [Multiple sclerosis and physical training]. *Ugeskr Laeger* 2005;167(14):1495-1499.

(8) Le Page C, Ferry A, Rieu M. Effect of muscular exercise on chronic relapsing experimental autoimmune encephalomyelitis. *J Appl Physiol* 1994;77(5):2341-2347.

(9) Rossi S, Furlan R, De C, V et al. Exercise attenuates the clinical, synaptic and dendritic abnormalities of experimental autoimmune encephalomyelitis. *Neurobiol Dis* 2009.

(10) Wens I, Broekmans T, Hendriks JJ, Savelberg HH, Hesselink MK, Eijnde BO. Muscle contractile properties and disease progress in EAE rats: impact of training intensity. *BMC Physiology* 2013;under review.

(11) Wens I, Dalgas U., Verboven K et al. Impact of high intensity exercise on muscle morphology in EAE rats. *Physiol Res* 2014;under review.

(12) Wens I, Verboven K, Kosten L, Grevendonk L., Dalgas U., Eijnde BO. Impact of high-intensity exercise on muscle contractile properties in experimental autoimmune encephalomyelitis (EAE) rats. *Mult Scler* 2013;19(11):311-312.

(13) Kent-Braun JA, Ng AV, Castro M et al. Strength, skeletal muscle composition, and enzyme activity in multiple sclerosis. *J Appl Physiol* 1997;83(6):1998-2004.

(14) Garner DJ, Widrick JJ. Cross-bridge mechanisms of muscle weakness in multiple sclerosis. *Muscle Nerve* 2003;27(4):456-464.

(15) Dalgas U, Stenager E, Jakobsen J, Petersen T, Overgaard K, Ingemann-Hansen T. Muscle fiber size increases following resistance training in multiple sclerosis. *Mult Scler* 2010.

(16) Souza-Teixeira FD, Costilla S, Ayan C, Garcia-Lopez D, Gonzalez-Gallego J, Paz JA. Effects of Resistance Training in Multiple Sclerosis. *Int J Sports Med* 2009.

(17) Albright A, Franz M, Hornsby G et al. American College of Sports Medicine position stand. Exercise and type 2 diabetes. *Med Sci Sports Exerc* 2000;32(7):1345-1360.

(18) Hawley JA, Lessard SJ. Exercise training-induced improvements in insulin action. *Acta Physiol (Oxf)* 2008;192(1):127-135.

(19) Wens I, Hansen D, Verboven K et al. The impact of 24 weeks resistance and endurance exercise on glucose tolerance in persons with multiple sclerosis. *Am J Phys Med Rehabil* 2014;epub ahead of print.

(20) Brooke MH, Kaiser KK. Muscle fiber types: how many and what kind? *Arch Neurol* 1970;23(4):369-379.

(21) Harris RC, Hultman E, Novikova NA. Glycogen, glycolytic intermediates and high-energy phosphates determined in biopsy samples of musculus quadriceps femoris of man in rest. *Scand J Clin Lab invest* 1974;33:109-120.
